# Chemical composition, anticancer and antibacterial activity of *Nepeta mahanensis* essential oil

**DOI:** 10.1186/s12906-022-03642-w

**Published:** 2022-06-25

**Authors:** Mahla Amirzadeh, Sara Soltanian, Neda Mohamadi

**Affiliations:** 1 Department of Biology, Faculty of Science, Yazd University, Yazd, Iran; 2 Department of Biology, Faculty of Science, Shahid Bahonar University of Kerman, P.O. Box 7616913439, Kerman, Iran; 3 Herbal and Traditional Medicines Research Center, Kerman University of Medical Sciences, Kerman, Iran

**Keywords:** *Nepeta mahanesis*, Essential oil, Anti-cancer, Oxidative stress, Antibacterial activity

## Abstract

**Background:**

Conventional cancer treatments, such as chemotherapy, radiation therapy, and surgery, often affect the patients’ quality of life due to their serious side effects, indicating the urgent need to develop less toxic and more effective alternative treatments. Medicinal plants and their derivatives are invaluable sources for such remedies. The present study aimed to determine the chemical composition, anticancer and antibacterial activities of *Nepeta mahanesis* essential oil (EO).

**Methods:**

The chemical composition of EO was analyzed by gas chromatography-mass spectrometry (GC-MS). Cytotoxicity and apoptosis/necrosis induction of EO was analyzed by MTT assay and Flow cytometry. Real-time PCR was performed to evaluate the *Bax/Bcl2* gene expression. Also, the effect of the EO on the cells’ mitochondrial membrane potential (MMP) and ROS level was assessed. DPPH assay was done to assess the free radical scavenging activity of the EO. The Antimicrobial activity, MIC, and MBC of the oil were determined via well-diffusion and broth microdilution methods.

**Results:**

Based on the GC-MS analysis, 24 compounds were identified in the EO, of which 1,8-cineole (28.5%), Nepetalactone (18.8%), germacrene D (8.1%), and β-pinene (7.2%), were the major compounds. Also, the EO showed considerable cytotoxicity against MCF-7, Caco-2, SH-SY5Y, and HepG2 after 24 and 48 h treatment with IC_50_ values between 0.0.47 to 0.81 mg/mL. It was revealed that this compound increased the *Bax*/*Bcl2* ratio in the MCF-7 cells and induced apoptosis (27%) and necrosis (18%) in the cells. Moreover, the EO treatment led to a substantial decrease in MMP, which is indicative of apoptosis induction. A significant increase in ROS level was also detected in the cells following exposure to the EO. This compound showed strong DPPH radical scavenging activity (IC_50_: 30). It was also effective against Gram-positive *E. faecalis* (ATCC 29,212) and Gram-negative *E. coli* (ATCC 11,333) bacteria.

**Conclusions:**

The results of this study demonstrated that the EO of *N. mahanesis* could be considered a bioactive product with biomedical applications that can be used as an alternative cancer treatment and applied in the biomedical industries.

## Background

Nowadays, because of the lack of definitive cancer treatment and the development of drug-resistant tumors, cancer prevalence has increased overwhelmingly, affecting all aspects of human life worldwide. Breast, lung, colon and rectum, and prostate are the most common cancers, respectively. Accounting for nearly 10 million deaths in 2020 worldwide, it is expected that the total number of cancer cases will rise from 19.3 million in 2020 to 30.2 million in 2040 [1, 2]. These figures highlight the argent for searching for effective alternative treatments.

Recently, medicinal plants and their derived products have been considered a valuable source of pharmacological and therapeutic agents with various biological activities [3, 4]. Plants’ extracts and essential oils (EOs) are complex mixtures of volatile and natural bioactive compounds representing antioxidant, anti-inflammatory, antimicrobial, and antifungal activity. They are used in pharmaceutical, food, cosmetics, and biomedical industries [5–7]. Moreover, these compounds have been shown to possess anticancer activity and the ability to reduce the side effects of commonly used chemotherapy drugs [8, 9]. Despite these advances, there are many unknowns in the way of establishing alternative therapeutic methods based on herbal medicines. That is why many types of research have been focused on medicinal plants and their anticancer properties.

*Nepeta L*. belongs to the subfamily Nepetoideae and the family Lamiaceae with about 300 species distributed in central and southern Europe, the near East, and central and south Asia. Many species of this genus, including 76 species, are endemic to Iran [10]. *Nepeta mahanesis* Jamzad & Simmonds is one of the Iranian endemic species. In Iranian traditional medicine, the decoction of aerial parts of this plant has been used as a sedative and for treatment of rheumatism, high blood pressure, stomach ache, and bone pain [11, 12]. However, a few studies have been conducted on the chemical composition and bioactivity potential of EOs of different species of *Nepeta* [13, 14]. To the best of our knowledge, no study has yet been done on the biological activities of *N. mahanesis* Essential oil (EO).

In the present study, we determined the chemical composition of *N. mahanesis* EO. We also evaluated the antibacterial activity and cytotoxicity of this compound against different cancer cell lines. Furthermore, the incidence of apoptosis and necrosis and the expression of *Bax*/*Bcl-2* genes in the cells were analyzed. In addition, the EO’s DPPH scavenging activity, as well as its effect on the reactive oxygen species (ROS) production and mitochondrial membrane potential, were assessed.

## Methods

### Plant sample collection

*N. mahanesis* sample in the full flowering stage (June to July 2019) was collected from Mahan city, Kerman province, Iran, and was authenticated according to the standard keys by Dr. Neda Mohamadi. The voucher specimen was deposited in the Herbarium Center of the Faculty of Pharmacy, KUMS (KF1423).

### Essential oil extraction

To prepare the plant’s essential oil, they were dried in the shade for 7 days. Next, about 100 g of the plant sample was macerated in water for 24 h before distillation to increase the penetration of the water. Hydrodistillation was done using a Clevenger-type apparatus for 3 h. The oil was separated from water, dried over anhydrous sodium sulfate, and stored in sealed vials at 4 ^º^C until used in further analyses and tests [15]. To prepare the EO for each test, the intended amount was weighted and solved/dispersed in the culture medium (for cell treatment) or deionized water (for DPPH).

### GC/MS analysis

GC-MS analyses were carried out on a Varian 3400 GC-MS system equipped with an HP-5MS (60 m × 0.25 mm i.d.); oven temperature was 50–265 °C at a rate of 5 °C/min, transfer line temperature 260 °C, carrier gas helium with a linear velocity of 31.5 cm/s, split ratio 1:50, ionization energy 70 eV; scan time 1 s and mass range 40–550 amu. The percentages of compounds were calculated by the area normalization method without considering response factors. The components of the oil were identified by comparison of their mass spectra with those of a computer library or with authentic compounds and confirmed by comparison of their retention indices either with those of authentic compounds or with data published in the literature. The retention indices were calculated for all volatile constituents using a homologous series of *n*-alkanes [16].

### Cell lines and cell cultures

The cytotoxic activity of *N. mahanesis* EO on human cancer cell lines, MCF-7 (breast adenocarcinoma), Caco-2 (colorectal adenocarcinoma), HepG2 (hepatocellular carcinoma), and SH-SY5Y (neuroblastoma), was evaluated by the colorimetric MTT (3-(4, 5-dimethyl thiazol-2yl) 2, 5-diphenyl tetrazolium bromide; Atocel, Austria) assay. All cell lines were purchased from the Pasteur Institute of Iran, Tehran. Iran. Cells were cultured in Dulbecco’s Modified Eagle’s Medium (DMEM) supplemented with 10% Fetal Bovine Serum (FBS) (Biowest, France) and 1% penicillin /streptomycin (Biowest, France). Incubation was carried out at 37 °C with an atmosphere of 5% CO2 and 95% humidity.

### MTT assay

The MTT assay was used to evaluate the cytotoxic effect of EO on cultured cells [17]. Briefly, 8000 cells/well were seeded in 96-well plates containing 100 µl of culture medium and incubated overnight. The medium was then replaced with a fresh medium containing different concentrations of the *N. mahanesis* EO (0.078, 0.0156, 0.312, 0.625, 1.25, 2.5 mg/mL). Wells treated with culture medium only considered as the control. After 24 and 48 h of treatment, 20 µL of MTT solution (5 mg/mL) was added to each well and incubated for 3 h. Then, the medium was discarded, 100 µL of DMSO was added to each well, and the absorbance of each well was measured at 570 nm using a microplate reader (BioTek-ELx800, USA). Finally, the percentages of cell viability were determined using Eq. 1. the obtained data were used to determine the concentration of the EO required to kill 50% of the cells (IC_50_) [18].

1 $$Cell\;viability\;(\%)\;=\;(Absorbance\;of\;treated\;cells/Absorbance\;of\;control)\;\times\;100$$

### Gene expression assessment

The expression of *Bax* and *Bcl-2* genes in the MCF-7 cells was assessed using real-time PCR. Toward this end, total RNA was extracted from untreated and EO- treated MCF-7 cells using the RNA isolation kit (DENA zist Asia, Mashhad, Iran) according to the manufacturer’s protocol. Next, complementary DNA (cDNA) was synthesized from total RNA using M-MuLV reverse transcriptase (Cat.No. EP0441; Thermo Scientific, Wilmington, USA) according to protocol. In the next step, the quantitative analysis of the expression of *Bcl2* and *Bax* genes was performed using SYBR Green (Pars Tous, Mashhad, Iran) according to the manufacturer’s protocol. Gene-specific primers were designed using Oligo7 Primer Analysis Software. Beta-2-microglobulin (*β2M*) gene was used as the housekeeping gene to normalize the gene expression values. The sequence of primers and product length are presented in Table 1. The reactions were carried out in triplicate using the QIAGEN apparatus (Germany).

**Table 1:** Details of primers used in the study

Gene name	Sequence (5´ to 3´)	Product size (bp)	Accession number
BAX	F: CCCGAGAGGTCTTTTTCCGAG R: CCAGCCCATGATGGTTCTGAT	155	NG_012191.1
BCL2	F: CATGTGTGTGGAGAGCGTCAA R: GCCGGTTCAGGTACTCAGTCA	83	NG_009361.1
β2M	F: CTCCGTGGCCTTAGCTGTG R: TTGGAGTACGCTGGATAGCCT	69	NG_012920.2

Amplification conditions for all genes were: 95º C for 15 min, followed by 40 cycles of 95º C for 30 s, 60 º C for 30 s, and 72 º C for 30 s. At the end of the PCR process, to derive melting curves, the temperature was increased in steps of 1 °C for 10 s from 61 to 95 °C. The specificity of products in reactions was validated by analyzing the melting curve of the products and gel electrophoresis. The expression level of target genes in treated samples compared with that of controls was calculated using the 2^−ΔΔCT^ ratio [17, 19].

### Flow cytometry analysis

The percentage of MCF-7 cells undergoing apoptosis and necrosis after treatment with *N. mahanesis* EO was measured by using the Annexin V-FITC/PI apoptosis detection kit (BD Biosciences) according to the manufacturer’s instructions.

Briefly, MCF-7 cells were seeded in 60 mm cell culture dishes. When the cells grew to 80% confluency, they were treated with the medium containing 0.625 mg/mL of the EO for 24 h. Then, the cells were collected, washed twice with PBS, resuspended in 1 x binding buffer reaching the cell concentration of about 1 × 10^6^ cells/mL, and incubated with annexin-V-FITC (5 µL) and PI (10 µL) in the dark for 30 min at 4 °C, followed by immediate analysis by flow cytometry (Partec Cyflow, Sysmex) using FL1 and FL3 filters.

### Measurement of mitochondrial membrane potential

For quantitative measurement of MMP, 2 × 10^4^ MCF-7 cells/well were seeded in sterile 96-well plates and incubated overnight. After 12 h, cells were treated with various concentration (0.078, 0.156, 0.312, 0.39, 0.468 mg/mL) of the EO for 4 and 24 h. Next, Rh-123 (10 µM) was added in the dark 30 min prior to the termination of the experiment. Subsequently, the cells were washed with PBS (three-time) and analyzed immediately using a fluorescence plate reader (FLX 800; Bio-Tek). Untreated cells were considered as control. The fluorescence intensity of cells was quantified at an excitation wavelength of 485 nm and an emission wavelength of 538 nm. The results were expressed as the fluorescence percentage of control cells.

### Intracellular ROS assay

Dichloro-dihydro-fluorescein diacetate (DCFH-DA) assay was used to assess the levels of ROS in the cells treated with the EO following the method previously described [20, 21]. Briefly, 25 × 10^3^ cells/well were seeded in 96-well microplates and incubated for 24 h. Then, their medium was replaced with a medium containing 20 µM of DCFH-DA (Sigma-Aldrich, Germany). After 1 h incubation in humidified atmosphere (5% CO2, 37 °C), cells were exposed to various concentration of EO (0.375, 0.625, 2.5, 5, 7.5 mg/mL) or 1500 µM H_2_O_2_ as a positive control. After 4 h, fluorescence was measured at an excitation wavelength of 485 nm and an emission wavelength of 538 nm (FLX 800; BioTek). Results were expressed as the percentage of fluorescence intensity relative to untreated control cells.

### DPPH free radical scavenging assay

1, 1-diphenyl-2-picrylhydrazyl (DPPH) radical scavenging assay was used to determine the ability of the EO to free radicals scavenging. Donating electrons or hydrogen to DPPH can convert DPPH from purple color into yellow and the extent of the reaction depends on the hydrogen releasing capacity of the compound [22]. In detail, 50 µl of the EO at various concentrations (0.01, 0.05, 0.5, 1, 2, 4, 4, 8, mg/mL) was added to 150 µl of DPPH solution (0.04 mg/mL in methanol). The mixture was shaken thoroughly and left at room temperature for 30 min. The mixture’s absorbance was then measured on a spectrophotometer at 517 nm using a plate reader (BioTek-ELx800, USA). Ascorbic acid was used as a positive control. DPPH radical scavenging activity was calculated by the following Eq. 2. The concentration that causes a decrease in the initial DPPH absorbance by 50% is defined as IC_50_. This value is calculated from the graph plotting the inhibition percentage versus the EO concentrations [9].

2 $$\mathrm{DPPH}\;\mathrm{radical}\;\mathrm{scavenging}\;(\%)\;=\;\lbrack(Ac-\;As)/Ac)\;\times\;100\%\rbrack$$

“A_C_” is the absorbance of the control (only DPPH in methanol), and “A_S_” is the absorbance of the sample.

### Antibacterial assays

Antibacterial activity of the extracted EO from *N. mahanesis* was evaluated against Gram-positive *Enterococcus faecalis* (ATCC 29,212) and Gram-negative *Escherichia coli* (ATCC 11,333) bacteria according to the methods previously described with slight modification [23]. The bacterial strains were obtained from the Iranian Biological Resource Center, Tehran, Iran.

#### Agar well diffusion method

To perform the agar well diffusion method, *E. coli* and *E. faecalis* bacteria were first cultured on Muller Hinton Broth (MHB) and incubated at 37 ˚C for 18–20 h. Next, the overnight bacterial cultures turbidity was adjusted to the 0.5 McFarland standard and cultured on Muller Hinton Agar using sterile swabs. Then, wells (6 mm in diameter) were made on the medium with the help of gel puncture and 50 µl of the *N. mahanesis* EO with different concentrations (300, 600, 1200 mg/mL) were inoculated into each well, and plates were incubated at 37 ˚C for 24 h. Finally, the diameter of zones of inhibition was measured. Ciprofloxacin (25 mg/mL) was used as the positive control.

#### MIC and MBC test

After the well-diffusion test, the antibacterial activity of EO was evaluated by determining MIC (minimal inhibition concentration) and MBC (minimum bactericidal concentration). MIC was determined using the broth microdilution assay. In detail, a serial dilution of the EO (300, 150, 75, 37.5, 18. 75, 9.37, 4.68, 2.34, 1.17 mg/mL) was prepared in Mueller Hinton Broth medium in 96-well plates. Next, 20 µl of each overnight bacterium (1.5 × 10^8^ CFU/mL) was transferred to the wells, and plates were incubated at 37 °C for 24 h. Control wells were filled with only culture medium and bacteria. The microorganism growth was determined by measuring the wells’ optical density at 600 nm (OD_600_). The MIC was considered the lowest concentration of the compound that inhibited the growth of bacteria so that no turbidity could be seen in the culture medium by naked eyes. To determine the MBC, broth dilutions of the EO that inhibited the growth of a bacteria were cultured on the nutrition agar and incubated at 37 °C for 24 h. The MBC is the lowest concentration of the compound at which no growth was observed in plates [24].

### Data analysis

The experiments were performed in triplicate and data was expressed as mean ± standard deviation. The Independent Samples t-Test was used to compare the means of two independent groups (control/untreated cells and treated cells) to determine whether the means were significantly different or not. The differences between the means were considered significant for values of p < 0.05. The half-maximal inhibitory concentration (IC_50_) values were calculated using Graph Pad Prism version 6.00 (GraphPad Software, San Diego, CA, USA).

## Results

### Essential oil GC-MS analysis

The EO isolated by hydro-distillation from *N. mahanensis* was pale yellow to yellow liquid. The quality and quantity of the compositions of the oil are shown in Table 2, where the compounds are listed by order of their elution on the HP-5 column. In total, 24 compounds were identified in the *N. mahanensis* EO, accounting for 93% of the total oil composition. The main components of this oil were 1,8-cineole (28.5%), nepetalactone (18.8%), germacrene D (8.1%), β-pinene (7.2%), caryophyllene oxide (4.25%), 1,3,6-octatriene (4.1%) and Myrtenal (3.16%).

**Table 2:** GC-MS analysis indicating phytochemical compounds of essential oil of *Nepeta mahanensis*

Compound ^a^	RI ^b^	RT^c^	(%) Composition
α-Pinene	942	4.18	1.62
Camphene	953	4.32	1.05
Sabinene	972	4.75	0.82
β-Pinene	976	4.84	7.26
β -myrcene	991	5.17	0.81
p-Cymene	1014	5.77	0.42
1,8-Cineole	1020	6.04	28.52
1,3,6-octatriene	1058	6.75	4.12
Linalool	1098	7.45	1.74
α-Campholena	1102	8.04	1.02
Transe-Pinocarveol	1119	8.43	1.78
Transe-Verbenol	1123	8.65	2.19
Pinocarvone	1134	9.01	1.82
Trans-pinocarveol	1139	9.22	0.36
Terpinen-4-ol	1157	9.41	0.61
Myrtenal	1165	9.61	3.16
α-Terpineol	1169	9.79	1.82
Myrtenol	1175	12.27	0.28
α-copaene	1376	13.2	0.42
Trans-caryophyllene	1418	13.87	1.23
Nepeta lactone	1427	14.31	18.85
Germacrene D	1473	16.38	8.15
Gamma-cadinene	1513	17.55	0.71
Caryophyllene oxide	1556	17.61	4.25
Total			93.01

^a^ Compounds are listed in order of their retention time from the HP-5 column. ^b^ Retention indices in elution order from HP-5 column. ^c^ Retention time of compounds

### Anticancer potential of *N. mahanensis* essential oil

**Fig. 1:**
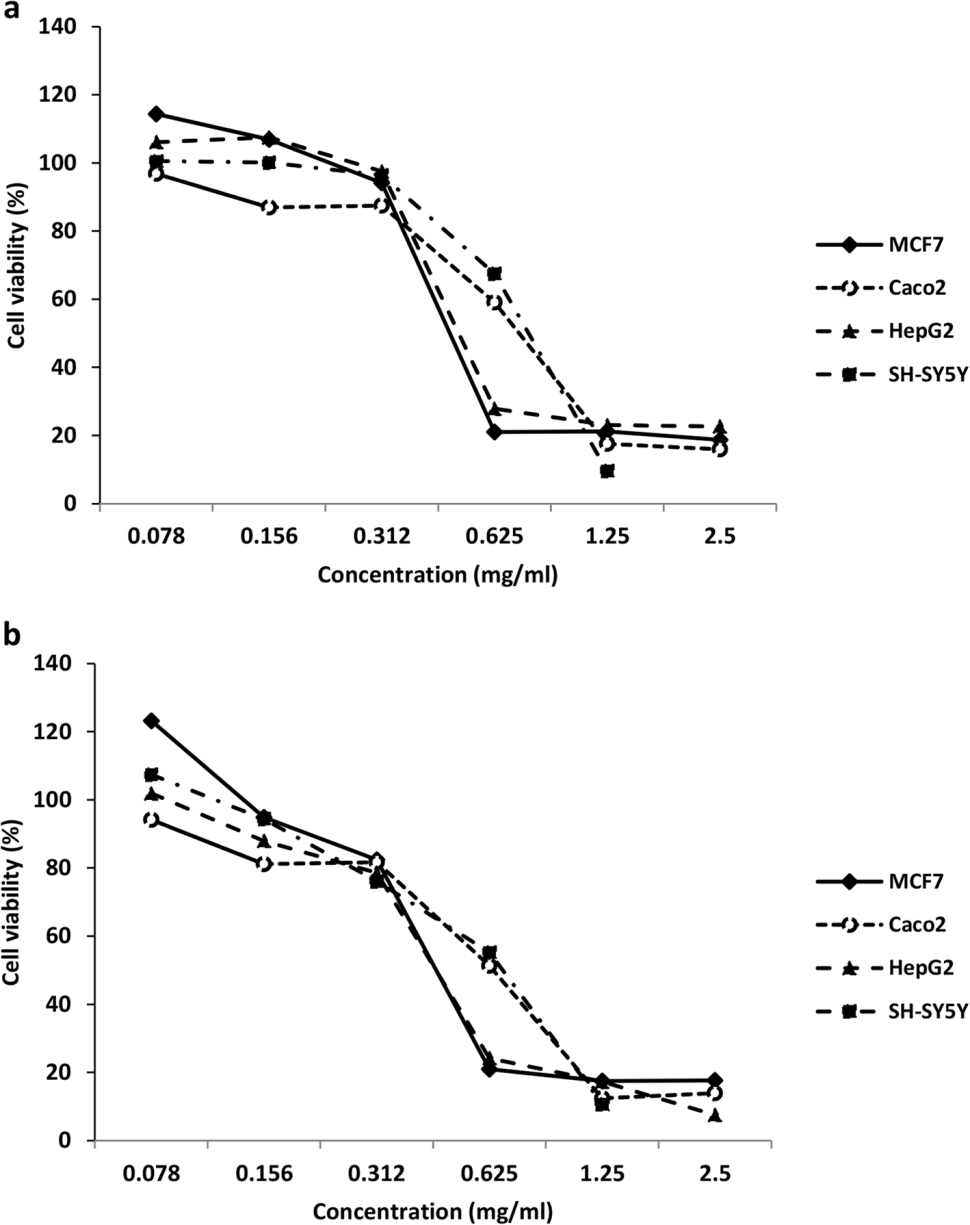
Cytotoxicity of *N. mahanesis* essential oils against a panel of four human cancer cell lines. Cancer cells were incubated with increasing concentrations of essential oil for 24 h (**a**) and 48 h (**b**). Estimation of cell viability was determined by the MTT assay

MTT assay results regarding the cytotoxicity of the EO against different human cancer cell lines, including MCF-7, Caco2, HepG2, and SH-SY5Y, are shown in Fig. 1a and b). Also, IC_50_ related to the 24 and 48 h exposure for all cell lines is summarized in Table 3. Based on the results, the EO showed a dose and time-dependent cytotoxic activity on all cell lines. This compound had the most cell toxicity against MCF-7, HepG2, Caco-2, and SH-SY5Y in the subsequent order. The viability of MCF-7 cells exposed to the EO decreased to less than 20% after 24 h.

**Table 3:** IC_50_ values of the *N. mahanesis* essential oil against cancer cell lines after 24 and 48 h of incubation

Cell lines	IC_50 (_mg/ml)	
	**24 h**	**48 h**
MCF-7	0.52	0.47
Caco2	0.76	0.65
HepG2	0.52	0.47
SH-SY5	0.81	0.70

### The expression of *Bax* and *Bcl2*

**Fig. 2:**
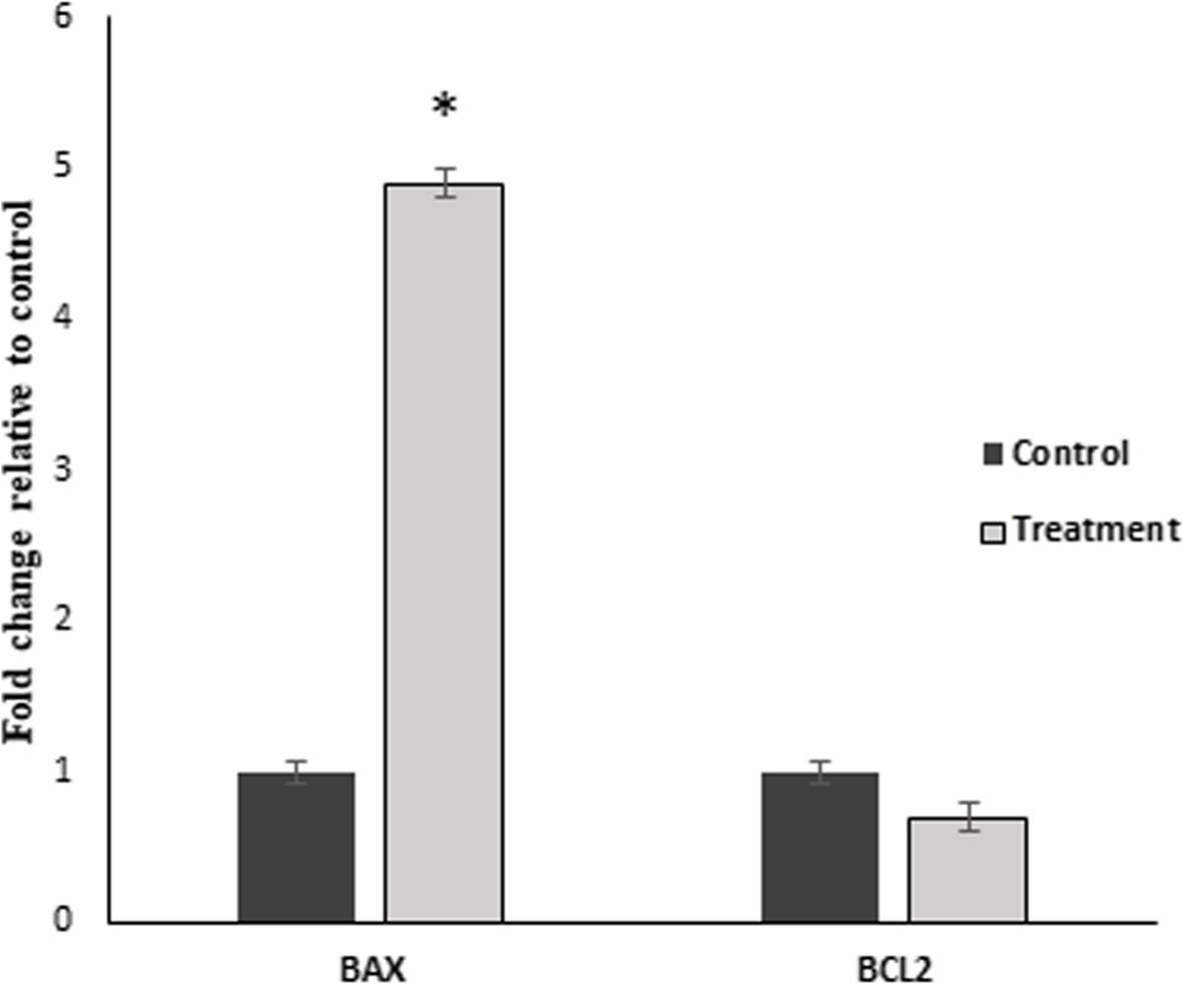
Evaluation of the effects of essential oil on the *Bax* and *Bcl2* expression level using qRT-PCR. MCF-7 cells were cultured and treated with essential oil (0.625 mg/ml) and *Bax* and *Bcl2* were examined in MCF-7 cells after 24 h of essential oil treatment. MCF-7 cells without treatment were used as a control to evaluate the relative expression of *Bax* and *Bcl2*. The relative gene expression of the control was set as 1. *p<0.05 shows significant differences as compared to the control  as tested by the student’s t-test

The expression levels of *Bax* and *Bcl2* were assessed in MCF-7 cells incubated with 0. 468 mg/mL of EO for 24 h, using qRT PCR. According to the results, no significant change in the expression level of *Bcl2* was observed. However, we observed that the mRNA level of *Bax* increased by 5 times (Fig. 2).

### Flow cytometry analysis

**Fig. 3:**
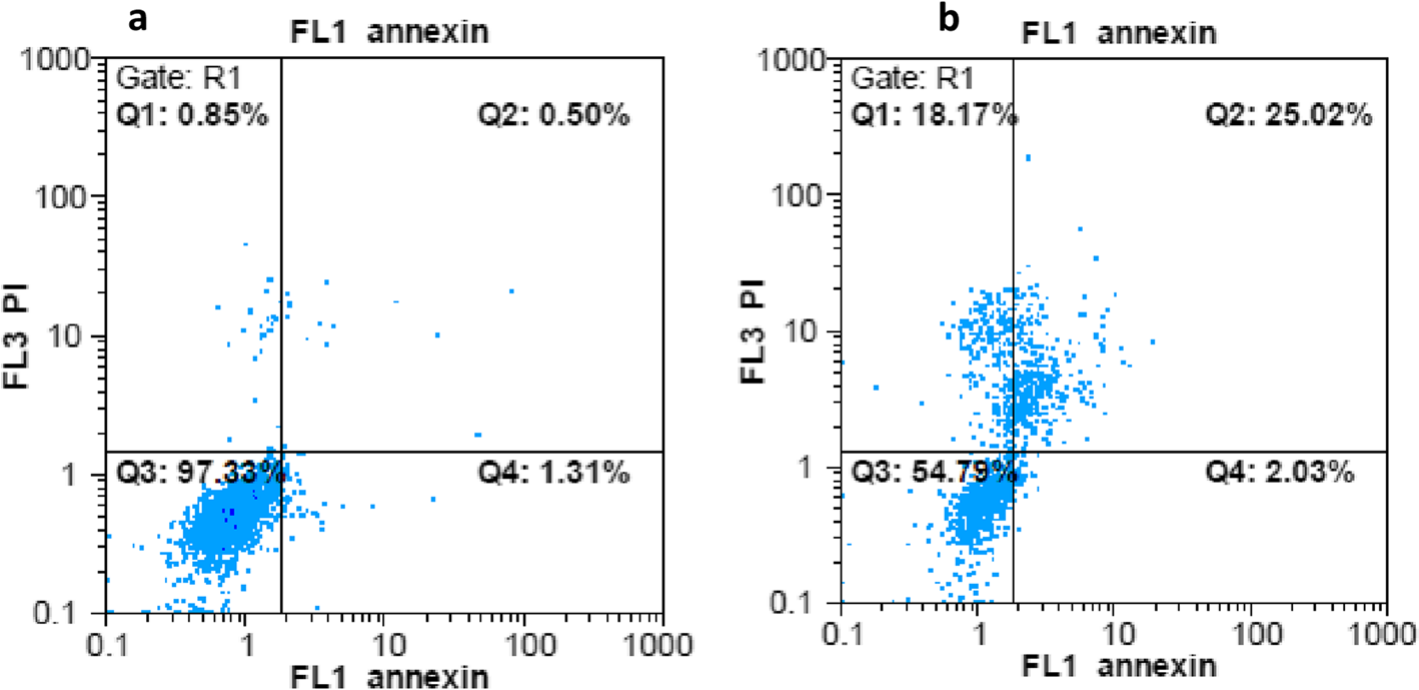
Cytotoxicity mechanism of *N. mahanesis* essential oils. Apoptosis and necrosis cells were evaluated using Annexin V-FITC/PI apoptosis detection kit in non-treated control cells (**a**) and essential oil -treated cells (**b**). Cells in the lower left quadrant are viable, those in the lower right quadrant are early apoptotic and those in the upper right and left quadrant are late apoptotic and necrotic

To determine the cytotoxic mechanism of the *N. mahanesis* EO, MCF-7 cells were treated with 0.625 mg/mL for 24 h. The incidence of apoptosis was monitored by flow cytometry after staining with FITC-annexin-V/PI. Figure 3 shows that the total percentages of PI-negative and annexin V-positive cells that were in early apoptosis and PI-positive and annexin V-positive cells that were in end-stage apoptosis are 27%. Moreover, this compound caused considerable necrosis (18%) in the treated cells.

### Mitochondrial transmembrane potential

**Fig. 4:**
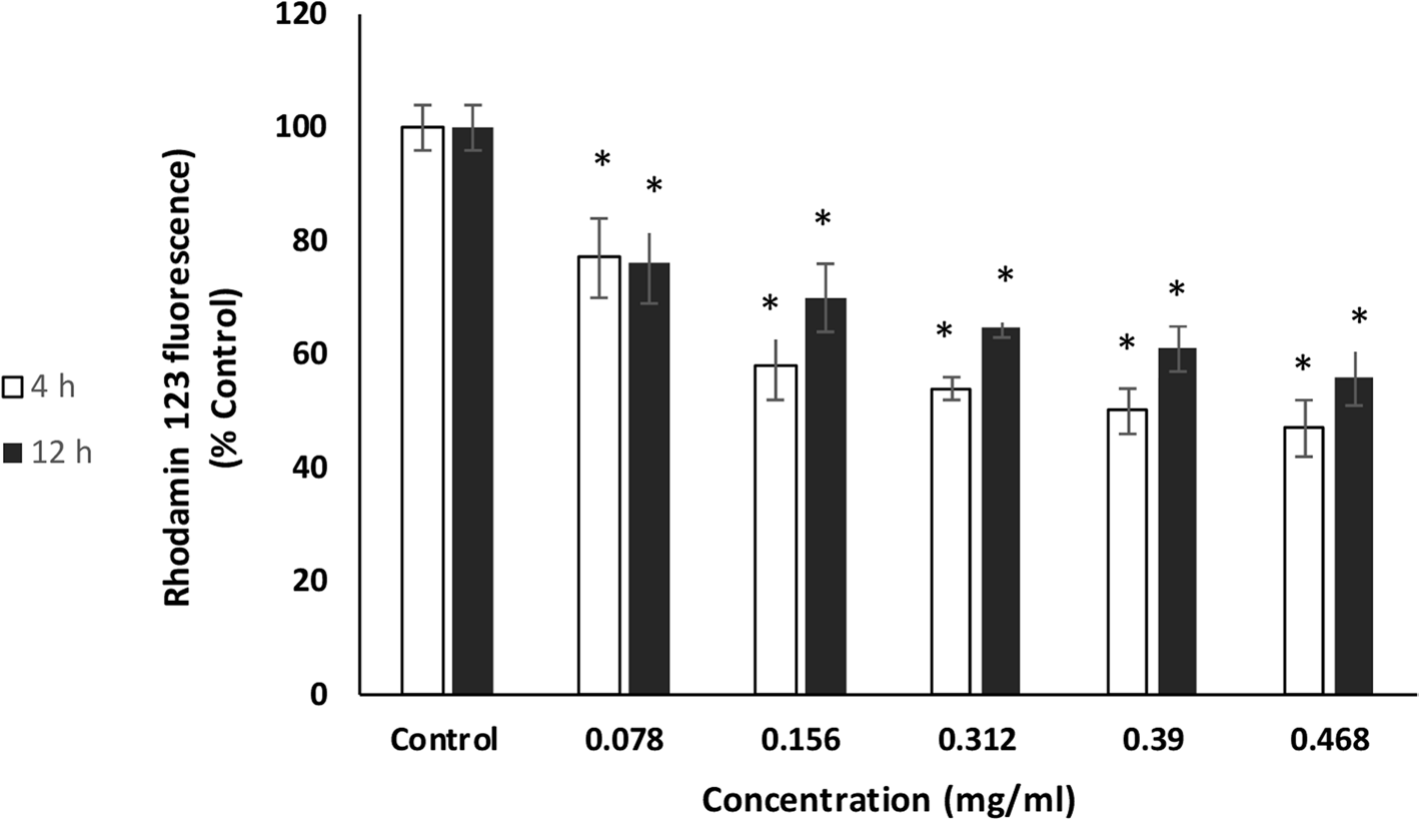
Effect of *N. mahanesis* essential oils on MMP in MCF-7 cells. Essential oil significantly reduced the MMP in a dose-dependent manner after 4 h and 12 h treatment. The results were expressed as Mean ± SD. *P<0.05 was regarded as significant difference in comparison to untreated control cells

The quantitative measurement of the MMP was done by treating the MCF-7 cells with EO concentrations ranging from 0.078 to 0.468 mg/mL. According to the results, untreated (control) cells did not show any change in ΔΨm, whereas ΔΨm level reduced significantly in MCF-7 cells following 4 and 12 h treatment with increasing concentration of *N. mahanesis* EO (Fig. 4).

### Intracellular ROS level

**Fig. 5:**
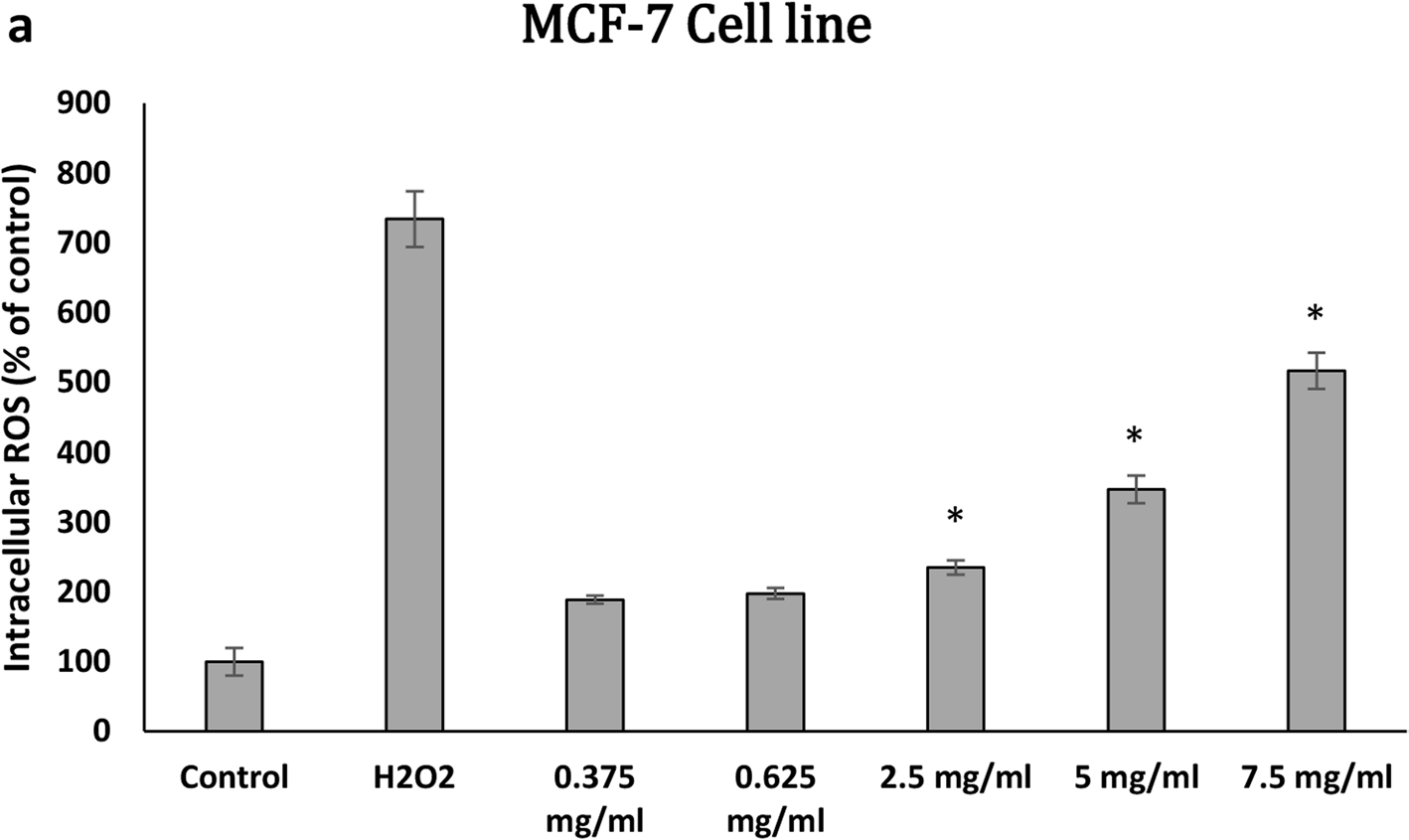
Effect of *N. mahanesis* essential oils on the formation of ROS. MCF-7 cells were treated with 1500 µM H2O2 as a positive control or different concentration of essential oil (0.375, 0.625, 2.5, 5, 7.5 mg/ml) for 4 h. Untreated cells were considered as control. Data are mean ±SEM of three independent experiments. *P<0.05 was regarded as significant difference in comparison to untreated control cells

The level of intracellular ROS in MCF-7 cells treated with the EO was assessed using the DCFH-DA assay. Our results showed that this compound at 2.5, 5, and 7.5 mg/mL concentration significantly induced oxidative stress and elevated the levels of ROS in a dose-dependent manner in the cell line. A 5- fold increased ROS level was observed in the cells treated with 7.5 mg/mL of EO (Fig. 5).

### DPPH radical scavenging activity

The DPPH assay suggested a concentration-dependent antiradical scavenging activity for the *N. mahanesis* EO. With the IC_50_ value of 30 µg/mL, the EO could inhibit the DPPH radical.

### Antibacterial activity

Results of the antimicrobial activity of *N. mahanesis* EO against gram-positive bacteria (*E. faecalis)* and gram-negative bacteria (*E. coli*) are shown in Table 4. As the results revealed, this compound had a considerable antibacterial activity against both bacterial strains. Inhibition zones for these bacterial strains were in the range of 7–11 mm, and MIC and MBC values were 2.27 mg/mL and 4.87 mg/mL for *E. faecalis* and 1.25 mg/mL and 2.27 mg/mL for *E. coli.*

**Table 4:** Antimicrobial activity of the *N. mahanesis* EO and its minimum inhibitory concentration (MIC), and minimum bactericidal concentration (MBC)

Bacterial Strains	Diameter of inhibition zone (mm)			MIC Value (mg/mL)	MBC Value (mg/mL)
	312 mg/ml	625 mg/ml	1250 mg/ml		
*E. faecalis*	9 ± 0.5	10 ± 0.4	11 ± 0.4	2.37	4.87
*E. coli*	7 ± 0.05	9 ± 0.5	10 ± 0.5	1.25	2.27

Data are expressed as mean ± SD of inhibition zone diameter (mm) for different concentrations of essential oil

## Discussion

Herein, we determined the chemical composition of *N. mahanesis* EO. We also demonstrated the cytotoxicity and antibacterial activity of this compound against different cancerous cells. Our results revealed that this compound, by increasing the *Bax*/*Bcl-2* and intracellular ROS as well as reducing the MMP, induced apoptosis and necrosis in MCF-7 cells. In addition, this compound could significantly inhibit the DPPH free radicals.

Our GC-MS results were in agreement with previous studies determining the chemical components of the *N. mahanensis* EO. F. Sefidkon et al. reported eighteen compounds in the oil of *N. mahanensis* and the major components were nepetalactone, 1,8-cineole, germacrene D, β-pinene, and caryophyllene oxide, in order of abundance [25]. These compounds were also identified in our study. It has been revealed that 4 different nepetalactone stereoisomers are major constituents of the EOs of plants belonging to the *Nepeta* genus [5, 26–28]. Moreover, the chemical composition of EOs extracted from other species of *Nepeta*, including *N. crispa, N. ispahanica, N. eremophila*, *N. ispahanica, N. menthoides* and *N. rivularis* were identified and 1,8-Cineole (11, eucalyptol), an achiral aromatic compound, was reported as one of the main components in all oils. This component is a terpenoid oxide with anti-microbial, anti-inflammatory, antioxidant, and anti-cancer activities [29, 30].

To date, many published articles have reported the anticancer activity of the plant extracts, EOs, and some of their isolated phytocompounds against various types of cancers. However, to the best of our knowledge, this is the first study on the *N. mahanensis* EO cytotoxic activity against cancer cell lines. Cytotoxic potential of the *N. mahanensis* EO can be attributed to the presence of phytocompounds, such as 1,8-cineole [31], β-Pinene [32], and β-caryophyllene oxide [33], which have shown promising cytotoxic and proapoptotic properties., Although nepetalactone and germacrene D constitute a high percentage of the *N. mahanensis* EO, there is no report on cytotoxic activity of nepetalactone and germacrene D. Noteworthy, EOs isolated from other *Nepeta* species such as *N. cataria* [34], *N. schiraziana* [35], *N. curvidens* [36], *N. sintenisii* [37], *N. sibirica* [27], *N. ucrainica* [38], *N. menthoides* [39] have been reported to exhibit cytotoxic effects against different types of human cancer cell lines. The results revealed that the EO obtained from *N. mahanensis* has potent cytotoxic effects on cancer cells and this activity is similar to another species of *Nepeta.*

Because of heterogeneous composition, various cytotoxicity mechanisms can be supposed for EOs, including induction of cell death by apoptosis and necrosis, cell cycle arrest, and disturbing key organelles’ function. EOs have low molecular weights and lipophilic nature that allow them to cross cell membrane leading to the disruption of cell membrane integrity and increased permeability.

Some reports showed a low concentration of EOs could induce apoptosis, whereas a high concentration of the compounds results in necrosis on cell lines [40]. Furthermore, it was shown that following treatment with cytotoxic compounds, cells undergo apoptosis at the earlier phase and eventually lead to necrosis [41]. In this study, we have shown apoptosis and necrosis induction of MCF-7 cells after treatment with EO with several assays, including analysis of mRNA expression level of *Bax* and *Bcl-2* gene, the measurement of MMP, and FITC-annexin-V/PI staining. All results showed apoptosis induction following treatment of cells with EO. However, some cells undergo necrosis after 24 h treatment with EO.

BAX and BCL-2 are the major members of the BCL-2 family that play key roles in regulating cellular apoptosis triggered by mitochondrial dysfunction [42]. In the present work, although the expression of *Bcl2* as an anti-apoptotic member of the BCL2 family did not change significantly, the *Bax* transcript was up-regulated following treatment of MCF-7 cells with EO, shifting the *Bax*/*Bcl-2* ratio in favor of apoptosis. These observations were confirmed by the results of Flow cytometry analysis, where the percentage of apoptosis increased considerably following cell treatment. The same result was obtained in a study by Khanzadeh et al. [43].

ROS generated in response to a variety of stimuli such as EOs can cause cell death via apoptotic or necrotic processes. Enhanced cellular levels of ROS can damage biomolecules such as proteins, DNA, and enzymes [44]. It has been demonstrated that ROS can trigger the release of cytochrome c from mitochondria and induction of apoptosis via a mitochondrion-dependent pathway [45].

In this study, we performed the DCFH-DA assay in order to determine whether the ROS production is involved in the cell death induced by *N. mahanesis* EO or not. The obtained results suggested that cell death caused by the *N. mahanesis* EO can be related to ROS production. Likewise, it has been reported that EOs extracted from other plants stimulated ROS production in a concentration and time-dependent manner that led to cytotoxicity against cancer cell lines [46, 47].

Our study showed that the *N. mahanesis* EO could strongly inhibit the free radical activity (IC_50_ = 30 µg/mL). In this regard, the EOs of different species of *Nepeta* has also been studied for their DPPH radical scavenging activity and their ability has been proven. The strong activity of the *N. mahanesis* EO agrees with the reported antioxidant activity of *N. faassenii* EO [48]. It is worth mentioning that the IC_50_ value obtained in this study is lower than those reported for the EO of *N. schiraziana* (IC_50_ = 52.24 µg/mL) [35] and *N. flavida* (IC_50_ = 42.8 µg/mL) [49], indicating the stronger radical scavenging activity of *N. mahanesis*.

The antioxidant activity of extracts and EOs obtained from plants can be attributed to the presence of terpenes and phenolic compounds [50–52]. In this case, strong in vitro antiradical activity of *N. mahanesis* EO is likely attributed to the relatively high content of the 8-cineole as a terpene [53].

Our study revealed the DPPH radical scavenging properties of the EO; meanwhile, this compound showed to induce ROS production in the cells. At first glance, it seems these two features are contradictory. But it should be taken into account that in the DPPH assay, the EO interacts with the DPPH, an organic chemical compound. It is a simple oxidation-reduction (redox) reaction that involves a transfer of electrons between two species. Here, the aldehyde groups of the EO phytocompounds play the electron donor role. At the same time, ROS accumulation is a complex/multifaceted process in the living cells exposed to the EO. It could be supposed that in such a circumstance, some of the EO components in one direction inhibit the free radicals. In contrast, others_ in various directions, such as hindering the enzymes’ functions and disrupting the electron transport chain_ increase ROS. Hence, it seems that these results are not contradictory.

Antibiotic-resistant bacterial infections are a serious problem that has prompted research into the identification of new drugs with antimicrobial activity, especially from natural sources including plants [54, 55]. In agreement with our study, previous studies on EOs driven from different Nepeta species demonstrated effective and broad-spectrum antibacterial effects against both gram-positive and gram-negative bacteria [56, 57]. The antimicrobial activity of EOs extracted from many plants such as *Nepeta* can be attributed to the high percentage of terpenoid compounds. Moreover, nepetalactones [58] and 1,8-cineole [30], as the main components of the EO, are responsible for this antimicrobial activity.

## Conclusions

In conclusion, to our knowledge, this is the first report on the biological activities of the *N. mahanesis* EO. The findings of the present study indicated that the *N. mahanesis* EO is rich in 1,8-cineole and nepetalactone. Given that 1.8 cineole has various biological activities including antibacterial and anti-cancer, therapeutic effects of EO can mostly be attributed to this component. The results of biological evaluations demonstrated that *N. mahanesis* has significant cytotoxic activity against cancer cell lines and possesses strong antibacterial activities. In addition, the cytotoxicity effect and necrosis/apoptosis-inducing action of *N. mahanesis* EO are mediated through increasing the *Bax*/*Bcl-2* genes’ expression and increased production of ROS and inducing oxidative stress. Also, the DPPH radical scavenging assay showed that EO could inhibit DPPH free radicals. Thus, the results of this study demonstrated the EO from *N. mahanesis* could be considered a bioactive natural product with the potential to be used as an alternative cancer treatment and applied in the biomedical industries.

## Data Availability

All data generated or analyzed during this study are included in this article and further details are available from the corresponding author on reasonable request. Accession numbers of analyzed genes are included in Table 1.

## Abbreviations

EO: Essential oil
ROS: Reactive oxygen species
GC-MS: Gas chromatography-mass spectrometry
MMP: Mitochondrial membrane potential
DPPH: 1, 1-diphenyl-2picrylhydrazyl
DCF-DA: 7-dichlorodihydrouorescein diacetate
Eos: Essential oil
MCF-7: Breast adenocarcinoma
Caco2: Colorectal adenocarcinoma
HepG2: Hepatocellular carcinoma
SH-SY5Y: neuroblastoma
MTT: 3-(4, 5-dimethyl thiazol-2yl) 2, 5-diphenyl tetrazolium bromide
DMEM: Dulbecco’s Modified Eagle’s Medium
FBS: Fetal Bovine Serum
qRT-PCR: Quantitative real-time reverse transcription-polymerase chain reaction
PS: phosphatidylserine
DCFH-DA: 2′,7′-dichlorodihydrofluorescein diacetate
DCF: 2′,7′-dichlorofluorescein
MIC: Minimal inhibition concentration
MBC: Minimum bactericidal concentration
ATCC: American Type Culture Collection
